# The complexity of shaping self‐management in daily practice

**DOI:** 10.1111/hex.12536

**Published:** 2017-02-02

**Authors:** Hester M. van de Bovenkamp, Jolanda Dwarswaard

**Affiliations:** ^1^ Institute of Health Policy & Management Erasmus University Rotterdam Rotterdam The Netherlands; ^2^ Research Centre Innovations of Care Rotterdam University of Applied Sciences Rotterdam The Netherlands

**Keywords:** autonomy, ethics, patient participation, qualitative research, self‐management

## Abstract

**Background and context:**

Many countries are giving patients a more active role in health care, on both the individual and collective level. This study focuses on one aspect of the participation agenda on the individual level: self‐management. The study explores self‐management in practice, including the implications of the difficulties encountered.

**Objective:**

To gain insight into the complexity of self‐management practice. This is crucial for developing both self‐management interventions and the participation policy agenda.

**Methods:**

Qualitative semi‐structured interviews with experts (n=6) and patients with a chronic condition (n=20).

**Results:**

In terms of level of involvement and type of activity, shaping self‐management in practice depends on personal and social dynamics, patients’ ideas of the good life and their interactions with care professionals. Clashes can arise when patients and professionals hold differing ideas, based on different values, about the level and type of patient involvement.

**Discussion:**

The discussion on self‐management should account for the fact that how we define self‐management is very much a normative issue. It depends on the norms and values of patients, professionals and underlying health‐care policies. Differing ideas present professionals with ethical dilemmas which they should reflect on. However, professional reflection alone is not enough to deal with these dilemmas. The participation agenda needs far wider ranging reflection on how participation relates to other values in health care.

## 1 INTRODUCTION

Many countries are giving patients a more active role in health care, on both the individual and collective level.^1^, ^2^, ^3^, ^4^ Individual patients are expected to act as health‐care consumers, critically choosing their health‐care provider and becoming active self‐managers of their disease.^2^, ^5^, ^6^ Collectively, patients or their representatives are asked to participate in all kinds of decision‐making processes such as guideline development, research agenda setting, quality improvement in health‐care organizations and government policymaking.^4^ There are high expectations for active participation. It would improve quality of care, give patients more autonomy and reduce public spending to name a few.^1^, ^2^, ^6^ Both scholarly and policy debates on patient participation place great emphasis on the positive effects. Indeed, participation can be considered a “hurrah word”—terms that evoke such a good image that they are hard to criticize.^7^, ^8^ The problem is that they receive too little critical reflection, while the literature shows that there is a great need for it.

Insights from the sociological, political science and public administration literature show that on both levels, participation practice is far more complex than policy documents often suggest. This raises important questions for health‐care professionals and policymakers alike.^1^, ^2^, ^3^, ^4^, ^8^, ^9^, ^10^, ^11^, ^12^, ^13^, ^14^ We want to build on the critical literature by focusing on one aspect of participation on the individual level: self‐management. This is an important pillar of the patient participation agenda in many countries,^1^, ^2^, ^3^, ^11^ and can be expected to become even more so in the future. For example, Huber et al.'s suggestion for a new definition of health as “the ability to adapt and self‐manage” can count on much attention.^15^ Because of the importance attached to the concept, it is vital to understand self‐management well and its possible consequences for health‐care practices.

The literature with a more critical stance towards the practice of self‐management points to several tensions. First, although self‐management is often proposed under “nice” labels such as patient autonomy, patient‐centred care and patient choice, it is on the neoliberal agenda to shift responsibility to citizens with the aim of reducing public spending.^2^, ^5^ This shift in responsibility has important consequences. For example, it may give patients the opportunity to become active but it also implies that they are to blame when they do not live up to the ideal and fail to self‐manage properly. Such focus on individual responsibility disregards the social context that determines whether and how patients can become active. Here, the way freedom is imposed on individuals can lead to patient abandonment and inequality.^2^, ^3^, ^11^, ^16^, ^17^, ^18^, ^19^ Second, despite the emphasis on self‐management and the creation of myriad interventions to support it, power relations remain firmly in place giving professionals the upper hand over patients who want to make their own decisions. This limits patients who want and have the capacity to become active.^12^, ^20^

The third aspect of self‐management practice that causes tension goes beyond the question of becoming active or not. It is about what the activity should entail. These tensions are closely connected to different interpretations of self‐management. While both academic and political debate commonly use the term self‐management, it is not a clear‐cut concept.^1^, ^2^, ^5^, ^21^, ^22^ The common denominator is “the involvement of patients in their own care process.” However, the extent and focus of involvement differ among definitions,^23^, ^24^ ranging from taking over medical tasks and following medical regimen to making autonomous decisions on living with a certain condition and dealing with all its emotional and social consequences.^2^, ^5^, ^21^ The various definitions have an important impact on the organization of care and division of responsibilities. For example, definitions that focus on taking over medical tasks and compliance to medical regimen, as professionals tend to use, grant power to the professionals and focus on medical outcomes.^1^, ^2^, ^20^, ^25^, ^26^ On the other hand, patients often have a holistic approach to self‐management and focus on living the good life. Managing their medical condition is part of that but there is more, causing patients to make choices that benefit their quality of life but go against the medical regimen.^2^, ^27^, ^28^, ^29^ The question then becomes how patients and professionals should deal with these differences in interpretation.

The tensions identified above show that the debate on self‐management is normative. It confronts health‐care professionals with ethical questions, such as what to do when patients cannot become active while this is expected of them or what to do when patients make choices that go against medical norms.^1^, ^25^, ^30^, ^31^, ^32^ The aim of this study was to combine and build on these critical insights by exploring the way patient self‐management is shaped in practice, including the implications of the difficulties encountered. This insight is important to develop self‐management interventions that recognize the complexities of self‐management practice. Moreover, insight into everyday experiences is crucial for the future development of the participation policy agenda.

## 2 METHODS

We conducted a qualitative study. First, we interviewed experts (n=6, Table 1), asking them to reflect on the concept of self‐management, the underlying values and how these might conflict with other values in health care. These respondents had expertise based on extensive experience as researchers in self‐management or medical ethics or as employees of organizations of professionals and patients. Previously, to gain insight into clinical practices, we interviewed nurses providing self‐management support (n=15). The results of these interviews are published elsewhere 1 and used as input for the interviews conducted with patients. We interviewed patients with chronic conditions (n=20, Table 2). In four cases, a family member took part in the interview. Respondents were contacted through the organization that provided their care. They were purposively selected on the criteria: (i) variation across medical conditions, (ii) variation across health‐care settings (outpatient hospital care, home care or a combination of these) and (iii) variation in ethnic background. The last criterion was considered relevant because cultural background may influence perceptions of self‐management.^18^, ^19^, ^20^, ^21^, ^22^, ^23^ The first and second criteria met the exploratory nature of the study and offered the opportunity to be sensitive to differences between conditions and settings that might arise from our data. Although this approach allows for this sensitivity, no firm conclusions about specific patient categories can be drawn because of the exploratory nature of the study.

**Table 1:** Interviewed experts

Expert	Role	Expertise
E1	Researcher and teacher	Nursing, ethics and self‐management
E2	Researcher	Patient participation, health‐care policy
E3	Researcher and teacher	Ethics and self‐management
E4	Ethics adviser of national nursing organization	Nursing, ethics
E5	Adviser patient organization	Patient participation
E6	Researcher	Health and self‐management

**Table 2:** Characteristics of interviewed patients and family members

	Chronic condition	Care provider	Gender	Country of origin	Additional remarks
Patient 1	Rheumatic disease	Hospital care	Female	The Netherlands	
Patient 2	Diabetes, kidney failure, glaucoma, gout	Hospital care	Female	The Netherlands	
Patient 3	Kidney transplantation	Hospital care	Female	The Netherlands	
Patient 4	Heart failure	Home care, hospital care	Female	The Netherlands	
Patient 5	Heart failure, hearing disability, vision problems	Home care, hospital care	Female	The Netherlands	
Patient 6	Cancer	Home care	Female	The Netherlands	
Patient 7	Tuberculosis	Home care, community care, hospital care	Female	Somalia	Nurse present
Patient 8	Tuberculosis	Home care, community care, hospital care	Male	Eritrea	Nurse present
Patient 9	Rheumatic disease, kidney failure, heart failure, hearing disability, immune disease	Hospital care	Male	The Netherlands	
Patient 10	Rheumatic disease, diabetes	Hospital care	Male	The Netherlands	
Patient 11	Rheumatic disease	Hospital care	Male	The Netherlands	
Patient 12	Kidney transplantation	Hospital care	Female	Morocco	Interpreter present
Patient 13	Kidney transplantation	Hospital care	Female	Morocco	Interpreter present
Patient 14	Rheumatic disease	Hospital care	Female	The Netherlands	
Patient 15	Rheumatic disease	Hospital care	Female	The Netherlands	
Patient 16	Kidney, hearing disability, high blood pressure, high cholesterol	Hospital care, home care	Female	Turkey	Interpreter present
Patient 17	Kidney transplantation	Hospital care	Male	Morocco	Interpreter present
Patient 18	Rheumatic disease	Hospital care	Female	The Netherlands	
Patient 19	Kidney transplantation	Hospital care	Female	Turkey	Interpreter present
Patient 20	Kidney transplantation, gastric bypass surgery, diabetes	Hospital care	Female	Turkey	Interpreter present
Partner 1	Patient 10		Female	The Netherlands	
Partner 2	Patient 12		Male	Morocco	Interpreter present
Partner 3	Patient 16		Female	Turkey	Interpreter present
Son	Patient 13		Male	Morocco	Interpreter present

The interviews with patients were semi‐structured, taking into account findings from the expert interviews and the earlier study on nurses’ experiences. We focused on patients’ experiences in managing a chronic condition, the patient‐professional relationship and the values adhered to (see Box 1). Interviews were conducted at the respondent's preferred location and took between 1 and 2 hours. In six cases, an interpreter was brought in because the respondents spoke little Dutch. The Erasmus MC Medical Ethical Committee approved the study (MEC‐2013‐350). All respondents provided informed consent to audiotape the interviews, and for us to use the data anonymously for scientific research.

Box 1 **Guide to patient interviews**

**Introduction**

- Respondent's background.
- Condition(s) respondent is suffering from, when did condition present itself, what type of care received.
- Impact of the condition on respondents life.

**Self‐management**

- Activities (now and in the past) to manage condition (medical, social, emotional).
- Relationship with professionals.
- Support received from health‐care professionals to manage condition.

**Values underlying good care**

- What is important in health care.
- Example of a good experience in health care.
- Example of a bad experience in health care.
- Influence experienced on care and treatment, evaluation of level of influence.
- Therapy adherence.
- Following professionals’ orders.
- Privacy.
- Access to care, health‐care costs.

Interviews were recorded and transcribed verbatim. Data analysis was a combination of induction and deduction. First, we openly coded our data. We then compared our codes to insights from the literature on self‐management. We paid specific attention to patients’ ideas of good care, how they related to self‐management and the possible conflicts or frictions that respondents reported as due to the differences in the ideas of patients and the professionals they encountered. This led to the following themes: (i) perceptions of self‐management, (ii) self‐management‐related activities, (iii) social factors influencing self‐management, (iv) self‐management in relation to living the good life, (v) professional‐patient relationship, (vi) frictions between professionals and patients and (vii) the implications of these frictions. To ensure reliability, the two authors discussed the themes until consensus was reached. The analytical themes were subsequently combined and analysed on how they influenced the way self‐management is shaped in practice both in terms of the level of activities (patients taking on a more active or passive role) and in terms of type of activity (in terms of patients focusing on their health or quality of life and how they choose to reach these goals for instance by making lifestyle changes or arranging the proper medical care). This led to the following three themes guiding our results section: (i) personal and social dynamics, (ii) ideas on the good life and (iii) interactions with health‐care professionals. During the last phase, the analysis was refined by the selection of poignant quotes illustrating for the various views and values of patients that provide clear insight into how self‐management is shaped in practice. To enhance the validity of the analysis, we discussed the findings with the advisory board of our study. They confirmed our findings and provided additional examples which helped refine our analysis. In an earlier phase of the project, the advisory board commented on the design and focus of the study. The advisory board was composed of patient representatives, researchers on self‐management and ethics and teachers in higher nursing education.

## 3 RESULTS

I think that self‐management is not a static concept. Self‐management depends on how much the disease influences my capacity. It depends on whether the disease is very active or in remission, on the support I get from my social network, and my normal capacity (…). And for me, self‐management means by definition that I get to decide what is important to me, what I want to hold onto, and how I can make the little changes that let me live a reasonably normal life. (P1)

This quote summarizes the complexity of self‐management in practice from the patient's perspective. Having a chronic condition means that one has to self‐manage living with that condition, no matter what. Not managing a chronic condition is not an option. However, the meaning of such self‐management differs between patients. In the following sections, we describe the complexity determining how self‐management of patients is shaped in practice, both in terms of (i) the *level* of involvement and (ii) the *type* of activity patients perform.

### 3.1 Shaping self‐management: personal and social dynamics

How self‐management is shaped partly depends on personal and social factors such as the skills patients possess, their social network and the stage of their disease. We will go briefly into them in turn.

First, respondents point out that to reach higher levels of involvement, one needs certain skills to be able to understand and process information and make decisions about one's health accordingly.

I see it in my own brother (…) [who also has diabetes]. He gets dizzy and doesn't feel well. I think this is because he's out of balance, because it's a complicated condition, you know. Because you have to eat every day and it's a balance between eating, medication and exercise. (…) I think managing diabetes, especially, it's not easy, not everyone can do it. (P2)

In particular, patients with an immigrant background feel that a language barrier prohibits self‐management in that it makes communicating what they want difficult, this can have a negative impact on their well‐being.

I get frustrated that I don't speak the language. I would've liked to point out this or that. There are days when we really get sick of it and feel really stressed. (P16)

Second, our respondents note that their social network is very important in shaping their self‐management. Family members sometimes take over tasks such as discussing their health with the professionals so that the patients can remain passive, as they wish. Other times, family members help patients play the active role they prefer. Besides influencing the level of involvement, the patient's social network can also shape self‐management by determining the type of activity patients perform, thereby influencing the choices patients make. For example, one respondent says that he is sticking to his disease‐induced diet because his wife decides what he can or cannot eat.

I'd love to sit down to a good roast, but that's not allowed, and now I don't get the chance [since my wife won't make them anymore]. (P10)

Third, respondents note that the extent to which one can play an active role is partly influenced by the stage of their disease. Sometimes respondents who would generally make their own decisions concerning their treatment are unable to play an active role because their illness stops them from doing so. In that case, they need professionals to take over.

When I'm not in pain, I'm empowered enough to make clear what I think. And believe me, I do that. But when you're in pain, you're not yourself. The understanding my doctor shows, when that happens, I really think she's one of the best. (P10)

Evidently, the way self‐management is shaped in practice not only differs between patients but also for individual patients at different stages.

### 3.2 Shaping self‐management: ideas on the good life

The way patients shape their self‐management is partly determined by their perception of the good life. Again, ideas of how to achieve the good life impact on the *level* of involvement and the *type* of patient activity.

For some, health is the most important aspect of living the good life. To manage their health, these patients prefer an active role in all aspects of care; they search for information about their condition, are proactive during medical consultations, make treatment decisions and consider how to integrate the chronic condition in daily life. One respondent with multimorbidity explained that she monitors her blood levels (eg, albumin), follows a special diet and explores whether different medications can be used together [without adverse effects].

People tend to think that I do too much and I'm always busy with it [managing her disease]. But then I think, What the heck, it's only because I do this that my kidney function is still okay, otherwise it would be much worse. My brother cared far less and he's already dead, even though he was 13 years younger than me. (P2)

This patient goes on to explain that having control over her condition makes her feel good. For example, she is glad when she can improve her blood levels.

The doctors doesn't make a fuss about it [her blood levels], but when I see that my levels are a little bit better than last time, then I'm happy with myself. (P2)

Some patients play a similar active role, but their view on the good life causes them to focus not so much on the medical aspects of their disease but on making decisions about their treatment in the light of being able to perform other social roles they find important. Having a chronic condition impacts on a wide range of issues in one's life which need to be balanced against one's health, such as the ability to work, participation in social events and making life decisions such as having children. Finding this balance sometimes implies non‐compliance with the prescribed medical regimen as the following example shows:

I was thinking about the example of one of the members of our board, who was on dialysis (…) and every time she went, she had to go on the dialysis machine for five hours. That was best for her. And then she said: ‘I am going to shorten that to four hours. It's not as good for me, I know that, but I also want to work and do this and that. Five hours just isn't an option for me.’ She'd decided to do something bad for her, medically speaking, but she said: ‘My well‐being is more important to me.’ (E5)

For other patients, quality of life means that they are less active self‐managers. They take their prescribed medication and speak up in medical consultations only when something is very important to them or when relatives push them to do so. However, generally, they do not play an active role in consultations, do not look for information about their condition or make adjustments to their medical regimen to fit their daily lives better. These patients value medical paternalism; they tend to follow doctors’ orders and expect professionals to decide in their best interests. They feel that the professionals know better than them and to play a more active role would limit their quality of life.

It's a conscious choice [not looking for information on the internet] because if I do a search, it will come up with lots of information, and that would drive me crazy. (P17)

Another respondent explained that she is “sensitive.” Her doctor recognizes this and does not burden her with information on her condition and treatment but tells her daughter instead. This approach protects her from suffering, she explains, which she values greatly.

But this doctor is really, how shall I put it, he's like a son. When I go for a consult he always takes trouble to really see me, he gives me my treatment and looks at the computer screen. And if something comes up, he'll tell my daughter and urge her not to tell me. […] (P19)

This quote already shows that the way self‐management is shaped in practice is further determined by the interactions patients have with their professionals. We turn to this subject next.

### 3.3 Shaping self‐management: interactions with health‐care professionals

Patients and professionals may share the same ideas of the preferred level of involvement in which case the professional can facilitate the preferred role. If desired, the professional will take decisions for patients who do not want to be involved in decision making, as we saw in the example above, or appreciate patients who are actively involved in decisions.

When I left, I said, ‘Thank you’ and he said, ‘Thanks to you too’. So I said, ‘What are you thanking me for?’ ‘Well’, he said, ‘the questions you ask make my work fun’. You know he has to think about things again. And I think that I did that on the dialysis ward too. Now and again I confronted people with the fact that they do their job in a certain way and that I muddle my way through that, as it were. (P3)

The respondents we talked to were generally satisfied with their involvement in the care they received. However, there were instances where patients’ and professionals’ ideas deviated, which affected the patients’ preferred level and type of involvement in several ways.

Firstly, patients do not always get the opportunity to play an active role in consultations with their health‐care professional.

In [hospital] I experienced someone telling me: “You'd better be quiet because we're the specialists”. I'd read something about a certain medication and asked if it could be something for me, and that was not appreciated at all. (P14)

Patients who want to play an active role often emphasize the importance of their autonomy: “Nothing happens to my body that I don't want to happen—that seems logical to me” (P10). This implies being allowed to make autonomous decisions, including choices that may not be the optimal medical option or one that they may regret later on. When patients make such decisions, this can have important consequences for the relationship with their professionals. One patient told how the professionals did not wish to continue the relationship after she rejected a proposed operation on her wrist:

Well, the orthopaedic surgeon was so domineering: “That has to be an operation”. I said: “I'm scared of complications […] for this and that reason”. Then no one in the team dared to support me. (…) They said: “We'll fix it later” but I said: “No thanks, I'll be suffering the consequences, not you.” (…) And then suddenly it was like: “Well, then you don't have any business here”. (P1)

Patients who expect professionals not to accept their choice will sometimes withhold information. One respondent who had consulted complementary medicine practitioners and had adjusted her medication based on their advice did not inform her doctor because she felt he would disagree. She feels that she is allowed to withhold this information. On the other hand, she feels that professionals are obliged to keep her informed.

It's my body, as the saying goes, you know, so he has to support me in what I find important for me; that's his job. (…) I think, yes, it's my life. I don't decide on his life, but he decides on mine. Then I think I should be informed about that. (P3)

Secondly, the reverse may apply when patients prefer to remain passive and professionals feel they should be active. Patients do not always appreciate being asked to make decisions.

What I don't understand is that the doctor sometimes asks me if I want to take certain medication or not. Isn't that the doctor's job? (P17)

Also, the professionals’ views on what patients can and should do for themselves can cause tension.

I asked this nurse to turn me over in bed now and again. But she only got angry because I was supposed to do that for myself. I said: “Why are you here then?” (P19)

Clashes like these can have important consequences for patients. One respondent was frustrated that he was discharged home quickly after a kidney transplant and had to take care of himself. This made him lose faith in the medical system.

My trust is gone. Even when someone says to me now: “I want to help you” I wonder if they really mean it. (P17)

Another respondent points out that inequality may be the effect of the current focus on activating patients.

Self‐management can take root in a whole system of people having to fight for themselves and then you get strong people having the upper hand and the weaker ones, who… (P1)

According to one expert, to prevent inequality, professionals need to tailor their support and cannot expect patients to be automatically active.

Equal access (…) in terms of what people can do, what they can understand and how well the provided information is aimed at people who can read at a high level. (…) We tend to forget that 20% of people have low literacy. How are you going to support them? (E3)

Thirdly, clashes on the active role of patients not only concern the preferred *level* of involvement but also the preferred *type* of involvement.

The doctor [GP] advised against an [kidney] operation time and time again. He kept insisting that I had to lose weight [first]. I tried really hard but I couldn't manage to lose weight. Eventually, because the doctors in the hospital said that it was time I had an operation, they decided on a gastric bypass. After a while I finally lost weight and I was given the go ahead for the operation. But if losing weight is so important, for my diabetes too, why didn't they propose doing a gastric bypass earlier? My doctor said maybe a thousand times that I had to lose weight, but I told him two thousand times that I couldn't. He told me that I was hurting my kidneys. Why did he wait until I had kidney damage? (P20)

In this case, the professional thought the patient should actively adopt a healthy lifestyle, while the patient felt she needed to be heard and that the professional should adjust his treatment accordingly. Another respondent relates how professionals thought he could self‐manage at home with the help of his family. He thought otherwise and became active by speaking up for himself and arranging for more professional care.

First they say: “You have kids, don't you? They can help.” And I say, yes, but the kids have jobs and should keep them and they also have their families; they need to be there for them as well. And if they also had to help their old man, it wouldn't work. That's not on, I don't want that. “But,” they say, “can't you move your bed downstairs?” Well yes, I can put a bed downstairs but in my room there will be no bed. I don't want that: lying there, looking out [at the world] from behind the geraniums on the windowsill; that's not going to happen. “Yes, yes, but don't you feel like cooperating?” So I say, what should I be cooperating with? I don't buy that; there are other options. (P9)

The above quotes again show the influence of personal/social dynamics and ideas on the good life. The factors discussed in our results are therefore intertwined in important ways.

In conclusion, the interactions between professionals and patients shape self‐management to an important extent. Different ideas on the preferred level and type of involvement can cause clashes between the two sides, which can impact negatively on the experienced quality of care. In the next section, we discuss what this and the other presented results mean for the discussion on self‐management and the broader participation agenda.

## 4 DISCUSSION

Many countries have placed patient participation on both individual and collective levels high on the agenda, with equally high expectations of what this participation can achieve. Confronted with the fact that participation is not yet delivering the right results, it is often concluded that the effort should be intensified to ensure that patients become equal partners in decision making.^12^ However, this conclusion does not do justice to the complex practices of participation.^2^, ^14^, ^33^, ^34^, ^35^ This study add insights into this complexity by focusing on how self‐management is shaped in practice.

As said in the Introduction, there are many definitions of self‐management.^21^, ^36^, ^37^ This is not just a theoretical discussion. Our analysis shows that in terms of both level and type of involvement, self‐management is shaped in practice and is influenced by a number of intertwined factors. First, self‐management is shaped by personal and social dynamics which are partly outside the patient's domain of influence. Skills, the social network and the stage of the disease all influence patient self‐management.^2^, ^3^, ^5^, ^38^, ^39^ It is difficult to generalize on the characteristics that might explain the differences between patients.^5^, ^14^ The only difference that stood out in our study is that patients with a non‐Western background report that the language barrier limits their playing an active role.^40^ Moreover, self‐management is very much shaped by patients’ ideas of the good life, which cannot be linked easily with personal characteristics either.^16^, ^26^, ^41^ For some patients, the good life means actively doing everything one can to remain healthy. Others are active too, but their health is not always the most important focus of their activities. For these patients, self‐management means doing things that medically speaking may not be the best option but offer a better quality of life.^27^, ^28^ For others, the good life means adopting certain self‐management tasks, such as taking medication, but assigning other aspects, such as decision‐making power, to the professionals.

The above points to the importance of professionals adjusting their care to the preferences and capabilities of their patients.^2^, ^5^, ^42^, ^43^, ^44^ Our respondents provided us with several examples of professionals enabling patients to play their preferred role. However, there are instances where professionals and patients hold differing ideas on the desired level and type of involvement. Patients wanting to make autonomous choices can clash with professionals who expect patients to follow their orders, while passive patients do not feel at ease with professionals eager to activate them. Clashes can also stem from differing ideas on the preferred type of activity. For instance, professionals may want their patients to adopt a healthy lifestyle while patients want to be able to speak up and choose medical options such as surgery. Thus, the interaction between professionals and patients further shapes self‐management in practice.

Although differences between patients have been identified before, this often results in well‐meaning recommendations to provide self‐management support according to the differences^23^, ^45^, ^46^ with a view to ensuring that all patients take on a far more active role. It is also concluded that professionals should change their ways and value patients input much more.^12^, ^47^, ^48^ Other researchers emphasize that not everyone is capable of being an active self‐manager to the same extent, and it should be recognized that some patients cannot perform an active role. Therefore, health‐care policies should not be based on the expectation that everyone can and wants to be active as this can cause inequalities between patients who can play such an active role and those who cannot.^2^, ^3^, ^11^ These recommendations seem to limit some of the problems identified in this study. However, our analysis shows that they do not do justice to the complexity of shaping self‐management. This discussion should go beyond dealing with the differences between active and passive patients. The differences reported in this study warrant a fundamental rethinking of the consequences of this discussion on self‐management.

The discussion on self‐management should account for the fact that how we define self‐management is very much a normative issue.^1^, ^3^ First, it depends on the norms and values patients adhere to. Whereas active patients stress the importance of their autonomy, less active patients value a paternalistic model of the professional‐patient relationship^49^ and trust professionals to make the right decisions. Second, it depends on the norms and values of professionals—including listening to patient preferences, following medical norms and preventing harm^1^—which can lead to different ideas on the preferred level and type of involvement. Third, professional‐patient interaction does not exist in a vacuum. The way self‐management is shaped also depends on the norms and values that underlie health‐care policies. As said, such policies tend to focus on giving patients a more active role and more say in health‐care decision making.^1^, ^2^, ^3^, ^4^ However, policymakers have a particular interpretation of self‐management in mind^2^ which does not necessarily correspond with those of the patients’. Moreover, health‐care policies are built on other values, such as providing care according to medical guidelines, ensuring patient safety and restricting health‐care costs.^50^ These norms greatly influence the way patients can self‐manage their disease, as it raises the question to what extent they can make choices that go against medical norms and result in higher costs (think of the patient wanting a gastric bypass instead of losing weight by taking up a healthy lifestyle).

These differing values present ethical dilemmas that underscore the importance of ethical reflection on self‐management by health‐care professionals.^1^ Self‐management interventions should accommodate such reflection. However, a broader critical discussion on the participation agenda is also important. The complexity of self‐management practice resonates with the complexities of other types of participation, such as participation in guideline development, health‐care supervision and quality improvement.^33^, ^34^, ^35^ What participation is depends on a complex set of values and factors; conflicts can arise that make participation very complicated in such cases. Therefore, a discussion is needed on how participation relates to other values in health care, such as following medical guidelines, ensuring safety, caring for vulnerable patients and containing health‐care costs. Policymakers, professionals and other actors involved in health care should reflect on such issues. The results presented in this study would be useful input for this reflection.

## CONFLICT OF INTEREST

The authors declare that there is no conflict of interest.
